# Influence of Monosodium Glutamate on Astroglia of Rat Habenula

**DOI:** 10.3390/biom15081111

**Published:** 2025-08-01

**Authors:** Aleksandra Krawczyk, Karol Rycerz, Jadwiga Jaworska-Adamu, Marcin B. Arciszewski

**Affiliations:** Department of Animal Anatomy and Histology, Faculty of Veterinary Medicine, University of Life Sciences, Akademicka 12, 20-033 Lublin, Poland; aleksandra.krawczyk@up.lublin.pl (A.K.); wisia2000@tlen.pl (J.J.-A.); mb.arciszewski@wp.pl (M.B.A.)

**Keywords:** habenula, astrocytes, GFAP, S100β, MSG, glutamate

## Abstract

The habenula (Hb) of the epithalamus is formed of the medial (MHb) and lateral (LHb) parts. The improper functioning of the Hb may lead to depression and anxiety. The glutamate excitotoxicity is accompanied by astroglia reactivity and leads to the damage of nervous system structures. The aim of the study was to assess the influence of monosodium glutamate (MSG) administrated subcutaneously to rats in doses of 2 g/kg b.w. (I) and 4 g/kg b.w. (II), on astroglia in the MHb and LHb. Based on immunohistochemical reactions, the morphology, number of astrocytes immunoreactive for glial fibrillary acidic protein (GFAP-IR) and S100β protein (S100β-IR), and their surface area, perimeter, number and length of processes, and cytoplasmic-nuclear immunostaining intensity for the studied proteins were assessed. In the MHb of animals receiving MSG, especially at a high dose, hypertrophy and an increase in the number of GFAP-IR and S100β-IR cells were demonstrated. In the LHb, only hypertrophy of processes in S100β-positive glia was observed. The immunostaining intensity increased in GFAP-IR glia and decreased in S100β-IR cells only in animals from group I. The results revealed that astroglia respond to MSG depending on its dose and the Hb part. This different behavior of glia may indicate their different sensitivity and resistance to damaging factors.

## 1. Introduction

The habenula (Hb) is an even nucleus of the epithalamus in the central nervous system (CNS) located near the third ventricle of the brain. This area is divided into a smaller, compact, medial part (MHb) and a much larger, looser, lateral part (LHb). These parts differ not only structurally but also functionally. This area plays a key role in the regulation of emotional and motivational behavior due to the presence of numerous connections with dopaminergic and serotonergic structures. The Hb is involved in experiencing negative emotions during stress reactions or pain. In addition, it affects the behavior of the organism in response to unpleasant external stimuli and enables appropriate reactions to unfavorable environmental conditions. This area processes information about threats, which allows for avoiding and minimizing unpleasant experiences [1,2,3,4]. In humans, excessive stimulation of Hb neurons affects their behavior and can also lead to the development of mental disorders such as depression or anxiety. Depressive-like symptoms were also observed in animals in which abnormal activity of structures within the Hb was demonstrated [1,4,5,6,7,8].

A key role in proper functioning of the MHb and LHb is played by astrocytes involved in the control and maintenance of an appropriate perineuronal microenvironment [3,6,9]. Astroglia provide trophic and metabolic support for neurons. Astrocytes are star-shaped cells with numerous projections. Their perisynaptic endings demonstrate the activity of many different ion channels and receptors for inter alia cytokines, chemokines, growth factors, and neurotransmitters including glutamate (Glu), which use astrocytic transmembrane transporters. In addition, astrocytes can be a source of many neuroprotective substances that have a beneficial effect on neuronal survivability [10,11,12,13,14,15]. Astroglia dynamically respond to any changes in the surrounding microenvironment. In pathological conditions, astrocytes become reactive and their increased activity is observed. First, cells seek to compensate for harmful environmental changes. At a later stage, reactive astrocytes manifest increased synthesis of, among others, glial fibrillary acidic protein (GFAP), which is responsible for intermediate glial filament formation, and nuclear–cytoplasmic calcium-binding protein S100β. Then, permanently or long-term, morphological and functional changes are observed, manifested by hypertrophy of cell bodies and processes, as well as increased proliferation and migration of glia [10,13,15,16,17,18,19,20]. Astrocytes in different brain regions exhibit diverse morphological and functional properties and respond differently to damaging factors. These cells undergo various phenotypic changes that may have either protective effects or exacerbate damage, depending on the nature and severity of the injury [13,21,22]. Numerous studies have identified two subtypes of reactive astrocytes: A1, which present neurotoxic properties, and A2, which have a beneficial effect on surrounding structures. The A1 subtype was identified in the context of inflammation induced by intraperitoneal lipopolysaccharide injection, along with microglial activation In contrast, the A2 subtype was identified based on its activation under ischemic conditions. Both subtypes are characterized by elevated levels of GFAP, which persisted for at least one week following the damaging stimulus [23,24,25,26].

Reactive astrogliosis is observed in the course of many acute and chronic CNS diseases in which Glu-induced excitotoxicity plays an important role in their pathogenesis [15,16,17,18,27]. Glu is one of the main excitatory neurotransmitters in the mammalian nervous system. A persistent and high concentration of Glu in the extracellular space contributes to the increased or prolonged activation of N-methyl-D-aspartate receptors (NMDA-Rs) located in neurons and glia. As a result, an excessive increase in intracellular levels of calcium ions may occur in the stimulated cells. This consequently triggers numerous mechanisms and enzymatic pathways leading to the damage and even death of neurons and glia [12,28,29,30,31]. The phenomenon of excitotoxicity and its irreversible effects are observed in the course of many acute and chronic CNS diseases, including epilepsy, stroke, traumatic brain injury, Alzheimer’s disease, and Parkinson’s disease [28,29,30,32,33,34]. Disturbances of Glu homeostasis have also been demonstrated in the Hb in experimental models of depression [3,35].

Previous in vitro studies by other authors have shown that exposure of astrocytes to high concentrations of glutamate induces a range of morphological changes such as swelling, reorganization of the GFAP cytoskeleton, and an increase in cell size despite the absence of cell death [36]. In another model, it has been shown that glutamate, as an energy substrate, may lead to the development of astrogliosis demonstrating features such as increased GFAP expression and morphological changes [37].

Numerous studies have shown that the administration of the sodium salt of glutamic acid (monosodium glutamate, MSG) contributes to an increase in Glu levels in blood plasma, in the extracellular space of the CNS, and also causes morphological and functional changes, including the damage or even death of structures in many areas of the brain, both in young and adult individuals. The nature of these changes depends on the route of administration and the dose of MSG. In adult animals, the most significant changes are observed after parenteral administration of the compound, particularly by intraperitoneal or subcutaneous injection, in large doses [38,39,40,41,42]. The morphological and functional changes observed in numerous studies within the CNS of animals receiving MSG are most likely the result of the excitotoxic effects of glutamate. Therefore, this compound is widely used in various animal models of excitotoxicity [40,42,43,44,45,46,47,48,49]. In our previous studies, we demonstrated morphological changes in both astrocytes and neurons in various brain areas of adult rats receiving MSG subcutaneously. In addition, we observed differences in the immunoexpression of selected proteins in the studied cells [50,51,52,53]. Therefore, we hypothesize that the administration of MSG, as a possible source of Glu, may induce changes in astroglial reactivity within the Hb.

The aim of the study was to evaluate the effect of MSG on astroglia in the MHb and LHb of adult rats, based on the analysis of astrocytic reactivity indicators using antibodies against GFAP and S100β proteins.

## 2. Materials and Methods

The experiment was approved by the 2nd Local Ethical Committee for Animal Experiments in Lublin, Consent no. 7/2011 (15 February 2011). Fifteen male Wistar rats were used for the studies after reaching the age of 60 days and an average body weight of 250 g. Female rats were not included in the experiment, as sex steroid hormones are known to influence astrocyte morphology, potentially affecting the consistency and clarity of the results [54].The animals come from Mossakowski Medical Research Institute, Polish Academy of Sciences (5 Pawinskiego Str. 02-106 Warsaw, Poland). Throughout the experiment, the animals were kept in cages (12 h day/12 h night cycle, ambient temperature 20–22 °C, and 60% air humidity) with constant access to food and water. For the experiment, the males were randomly divided into three groups, i.e., control (Cont), I, and II, with 5 animals in each group. Then, for 3 consecutive days, the rats were given a subcutaneous (s.c.) injection of physiological saline (group Cont) and MSG (Sigma-Aldrich, St. Louis, MO, USA, 49621) in two doses: 2 g/kg b.w. (group I) and 4 g/kg b.w. (group II). The neurotoxic effect of MSG was confirmed in various research models [42]. On the fourth day of the experiment, all animals were euthanized and the brains were immediately collected and placed directly into a fixative solution (immersive fixation) immediately after extraction. No transcardial perfusion with saline or formalin was performed. Buffered 10% formalin (pH = 7.0, temp. 4 °C, 12 h) was used to fix the material. The brains were then embedded in paraffin blocks which were then cut into coronal 4 µm thick sections containing the Hb (A 4230 µm—A 3990 µm, according to a stereotaxic atlas) [55].

### 2.1. Immunohistochemical Analyses

Immunohistochemical reactions were performed using the indirect peroxidase–antiperoxidase method with antibodies from Sigma-Aldrich (St. Louis, MO, USA). According to the manufacturer’s recommendations, all reagents were diluted in 0.5M Tris buffered saline (pH = 7.6) which was also used to wash the sections. All Hb-containing brain sections from each animal were deparaffinized and rehydrated, then incubated with 3% H_2_O_2_ for 30 min, and then with goat serum (G9023, 1:10) for 20 min at room temperature (RT; 21 ± 1 °C). Subsequently, a primary rabbit anti-GFAP antibody (G 9269, 1:80) and a mouse anti-S100β antibody (S2532; 1:1000) were imposed on the slides for 16 h at 4 °C. After this time, the sections were washed and further incubated at RT for 1 h with a species-specific (rabbit (A9169, 1:400) and mouse (A9917; 1:200), respectively) secondary goat anti-IgG antibody conjugated to a peroxidase–antiperoxidase complex. Then, 3,3′-diaminobenzidine tetrachloride (DAB, 32750, Sigma-Aldrich, St. Louis, MO, USA) was used as a chromogen. In the next step, the sections were washed in distilled water and were counterstained with Mayer’s hematoxylin (MHS80, Sigma-Aldrich, St. Louis, MO, USA) according to the routine histological staining procedure. In parallel, a negative control reaction was performed by omitting the primary antibodies, the specificity of which was confirmed on rat brain sections in our previous studies [51,53]. For the morphological assessment of GFAP-immunoreactive (GFAP-IR) and S100β-immunoreactive (S100β-IR) cells in the MHb and LHb of rats, analyses of all stained sections containing the Hb were performed in an Olympus BX 40 light microscope with an Olympus Color View III digital camera (Olympus, Tokyo, Japan) using 40× and 60× objectives. Parameters such as light intensity, exposure time, and white balance were kept constant for all analyzed specimens, which ensured the comparability of images obtained from different experimental groups.

### 2.2. Morphological and Morphometric Analyses

During microscopic observations, microphotographic documentation was prepared, which allowed for a further morphometric assessment of the examined glia using the Cell^D program.

In both parts of the Hb of rats from groups Cont, I, and II, the number of GFAP-IR and S100β-IR astrocytes and the number of all cells were analyzed in 100 fields with an area of 2.0 × 10^−2^ mm^2^. Analyses were performed using a grid in randomly selected 20 fields from both parts of the Hb and from each animal (5 tissue samples per individual). The obtained results were presented as the average number with standard deviation of all cells and cells immunopositive for the examined proteins in the MHb and LHb.

In addition, the number and length of processes, area, perimeter, shape index (Si), and optical density (OD) of GFAP-IR and S100β-IR cells were analyzed in both examined parts of the Hb of rats from groups Cont, I, and II.

The number of primary astrocyte processes was counted in 50 random cells. The length of astrocytic processes was measured from the cell nucleus to the tip of the process by manually marking the shape of the process in 50 random astrocytes from every group of rats.

For the measurement of the cellular area and perimeter, an interpolated polygon tool was used to mark the bodies of 50 astrocytes. Based on these results, the Si of the tested cells was calculated using formula Si = 4π × A/P2, where A is the area and P is the perimeter of the cell body. Increasing index values represent rounder rather than oval cell bodies.

Optical density (OD) measurements, considered as a proxy for semi-quantitative evaluation of immunoreactivity, were conducted as described in previous studies [56,57]. The staining intensity was measured in a region of interest of 1 mm^2^ in 100 cells with brown reaction product. A total of 20 cells per animal were randomly selected from both the medial and lateral regions of the habenula (Hb), based on 5 tissue sections collected from each individual. The intensity of free space was measured to standardize the differences in light exposure. Moreover, the results were inverted so that higher values represented darker staining and lower values represented brighter colors. Hence, OD was counted as OD = 255 − (255x/y) where x is the measured intensity in the cell and y is the measured intensity of free space [56].

Additionally, thresholds were used to classify the intensity. Strong reaction was represented by results above OD = 195, moderate reaction with OD = 135−195, weak reaction as OD = 75−194, and results below 74 were considered as negative.

All results were presented as means with standard deviation.

### 2.3. Statistical Analyses

Statistical analyses were performed using Statistica 13.3 software (TIBCO Software Inc., 2017; Palo Alto, CA, USA). Results in the form of means with standard deviation were compared within groups between the tested parts of the Hb (MHb vs. LHb) and between the groups of animals (Cont and I, II) within both parts of the Hb. Normal distribution and homogeneity of variance were assessed using the Shapiro–Wilk and Levene’s tests, respectively. Results meeting the conditions of normal distribution and homogeneity of variance were compared using the one-way analysis of variance (ANOVA) test with the Tukey HSD post hoc test for multiple comparisons. Other results that did not meet these conditions were assessed using nonparametric tests. The Kruskal–Wallis test for multiple comparisons and the Mann–Whitney U test for single comparisons were used. The significance factor of all tests was set at α = 0.05.

## 3. Results

### 3.1. Morphological Analyses of GFAP-IR Astrocytes

In all studied groups of animals, astrocytes with GFAP-immunoreactive cytoplasm around round or oval cell nuclei and at the basis of the processes were demonstrated in the MHb and LHb.

**Figure 1:**
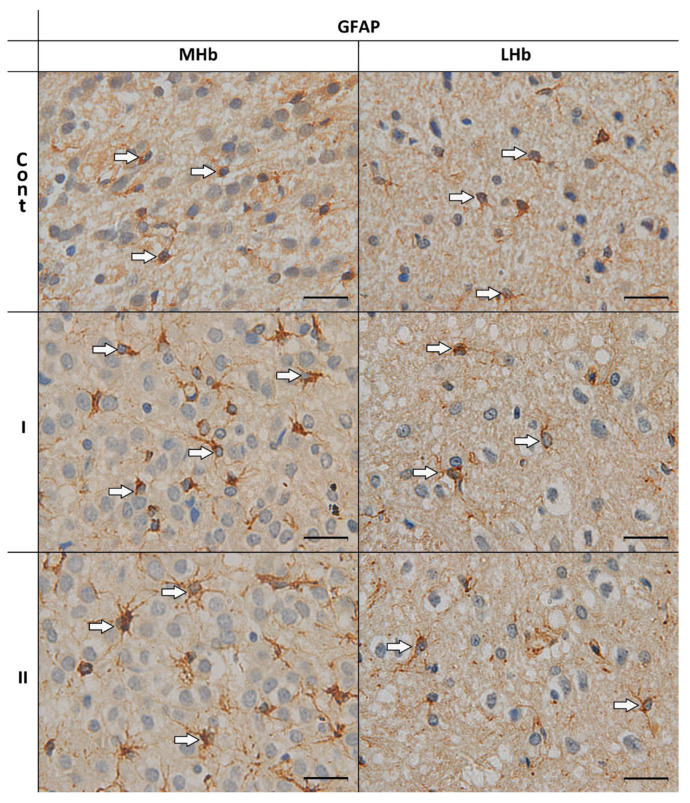
GFAP–immunoreactive cells in the MHb and LHb of control rats (**Cont**) and rats treated *s.c.* with MSG at a dose of 2 g/kg b.w. (**I**) and 4 g/kg b.w. (**II**). GFAP-immunoreactivity was detected as described in the Materials and Methods. The arrows indicate the astrocytes. Scale bar 20 µm.

In the MHb of control animals (Cont), cells with the presence of the studied protein mainly in the cytoplasm of the bodies and in the weakly branched processes were observed in the majority. In rats from group I, astrocytes were mostly characterized by GFAP-immunoreactive cytoplasm located at one pole of the cells. Thicker processes had few and thin branches. In the MHb of animals receiving MSG at a dose of 4 g/kg b.w. (II), astrocytes immunopositive for GFAP showed a high content of cytoplasm with immunoexpression of the tested protein in the body and in the thicker, branched processes radiating from the body (Figure 1).

In the LHb of all studied animals (Cont, I and II), GFAP-positive cells showed similar morphology. Mainly, astrocytes with immunopositive cytoplasm of the body were observed at one of the cell poles and in weakly branched processes (Figure 1).

### 3.2. Morphological Analyses of S100β-IR Astrocytes

**Figure 2:**
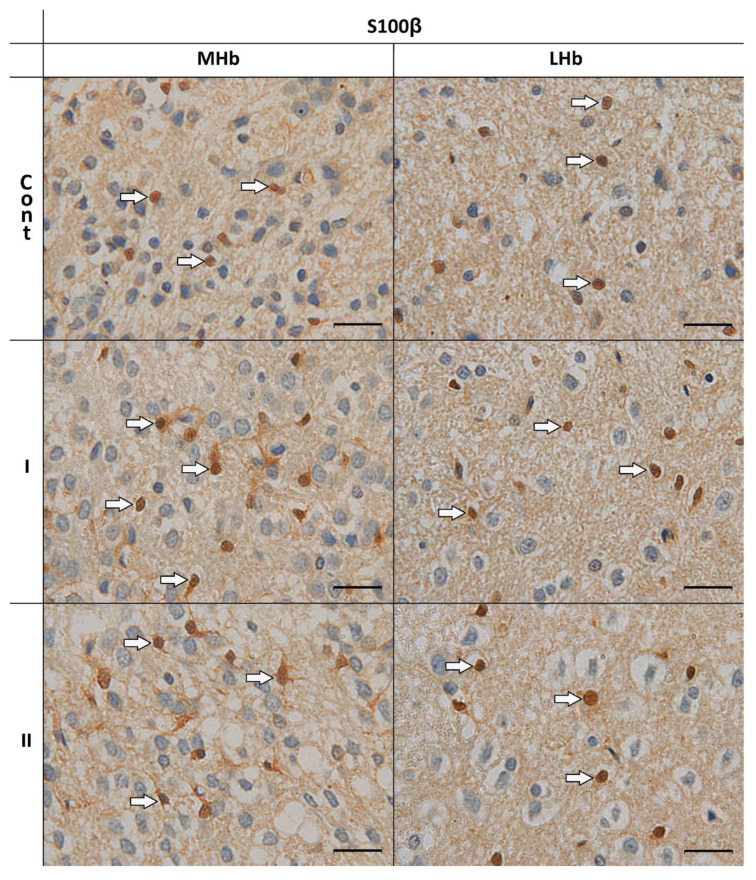
S100β–immunoreactive cells in the MHb and LHb of control rats (**Cont**) and rats treated *s.c.* with MSG at a dose of 2 g/kg b.w. (**I**) and 4 g/kg b.w. (**II**). S100β-immunoreactivity was detected as described in the Materials and Methods. The arrows indicate the astrocytes. Scale bar 20 µm.

In all studied rats (Cont, I, II), in the MHb and LHb, cells with immunoexpression of S100β in the cytoplasm of the bodies and in round or oval cell nuclei were demonstrated. Moreover, in both examined parts of the Hb in control animals (Cont), S100β-IR astrocytes were characterized by immunostained cytoplasm at the site of departure of short processes. There were also cells without processes. In rats from groups I and II, especially in the MHb, cells immunopositive for the studied protein were observed with longer and weakly branched processes originating from one pole of the body. Also, in these groups, some of the astrocytes were without processes (Figure 2).

### 3.3. Morphometric and Statistical Analyses

#### 3.3.1. Density of Cells

**Figure 3:**
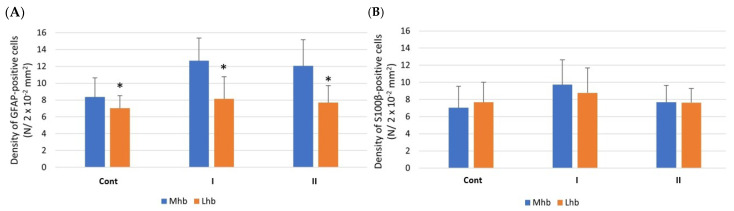
The density of GFAP-positive cells (**A**) and S100β-positive cells (**B**) in the MHb and LHb of control rats (**Cont**) and rats treated *s.c*. with MSG at a dose of 2 g/kg b.w. (**I**) and 4 g/kg b.w. (**II**). *, statistically significant differences, Mann–Whitney U test, *p* < 0.05.

In the MHb in all examined animals (Cont, I, II), the number of astrocytes with GFAP immunoexpression was statistically significantly higher in comparison to the LHb (Figure 3A). On the other hand, the number of S100β-positive cells in both examined parts of the Hb was comparable (Figure 3B).

**Figure 4:**
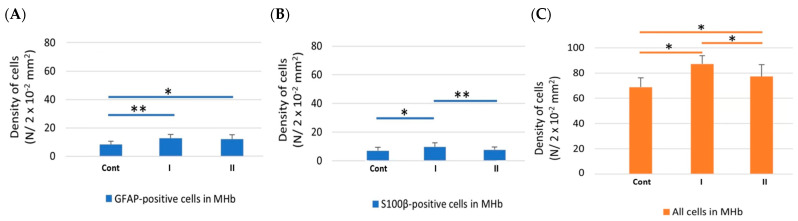
The density of GFAP-positive cells (**A**), S100β-positive cells (**B**) and all cells (**C**) in the MHb of control rats (**Cont**) and rats treated s.c. with MSG at a dose of 2 g/kg b.w. (**I**) and 4 g/kg b.w. (**II**). *, statistically significant differences, ANOVA, *p* < 0.05; **, statistically significant differences, Kruskal–Wallis, *p* < 0.05.

In the MHb in animals receiving MSG (I and II), the results indicated a statistically significantly higher number of astrocytes immunopositive for GFAP in animals receiving MSG at both doses (I and II) in comparison to control rats (Figure 4A). The number of cells with S100β immunoexpression was significantly higher in individuals from group I compared to animals from group Cont and II in which the density of the examined cells was comparable (Figure 4B). In addition, the total number of cells significantly increased in rats receiving MSG compared to the control group. In animals from group I, the number of immunopositive cells was higher than in both Cont and II groups of rats (Figure 4C).

**Figure 5:**
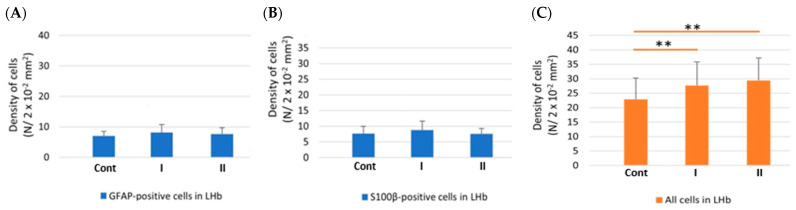
The density of GFAP-positive cells (**A**), S100β-positive cells (**B**) and all cells (**C**) in the LHb of control rats (**Cont**) and rats treated sc. with MSG at a dose of 2 g/kg b.w. (**I**) and 4 g/kg b.w. (**II**). *, statistically significant differences, ANOVA, *p* < 0.05; **, statistically significant differences, Kruskal–Wallis, *p* < 0.05.

In the LHb, analyses did not show significant differences in the density of astrocytes with GFAP immunoexpression and S100β-positive cells in individuals between all of the studied rats (Figure 5A,B). However, in rats from groups I and II, the total number of cells increased significantly in comparison to animals from group Cont (Figure 5C).

#### 3.3.2. Morphometric Parameters of GFAP-IR Astrocytes

Astrocytes with GFAP immunoexpression in the MHb and LHb in control individuals (Cont) had a comparable number and length of processes, as well as surface area and perimeter of the cell body. In contrast, in individuals receiving MSG (I and II), the values of these parameters were statistically significantly higher in the MHb compared to the LHb. Moreover, in the MHb in these animals (especially in group II), an increase in the number and length of GFAP-immunopositive processes, as well as the size of surface area and body perimeter of GFAP-IR astrocytes, was demonstrated compared to rats from group Cont. In contrast, in the LHb in all examined individuals (Cont, I, II), the values of the studied parameters were comparable (Table 1).

#### 3.3.3. Morphometric Parameters of S100β-IR Astrocytes

Analyses of the number and length of S100β-immunopositive processes and the size of the surface area and body perimeter of astrocytes with the immunoexpression of the studied protein showed statistically significantly higher values in the MHb compared to the LHb in all rats receiving MSG (I and II). In contrast, in control individuals, S100β-positive cells had statistically significantly more processes and a smaller surface area and body perimeter in the MHb compared to the LHb.

In the MHb, analyses of selected parameters showed their higher values in individuals receiving MSG (I and II) compared to control animals (Cont). Additionally, more S100β-IR processes extending from astrocytes were observed in rats from group II. In the LHb, in individuals from groups I and II, the cells had more numerous and longer processes with a brown reaction product compared to group Cont. However, the surface area and body perimeter values of S100β-positive astrocytes in all examined individuals (Cont, I, II) were comparable (Table 2).

#### 3.3.4. Immunostaining Intensity of GFAP-IR Cells

**Figure 6:**
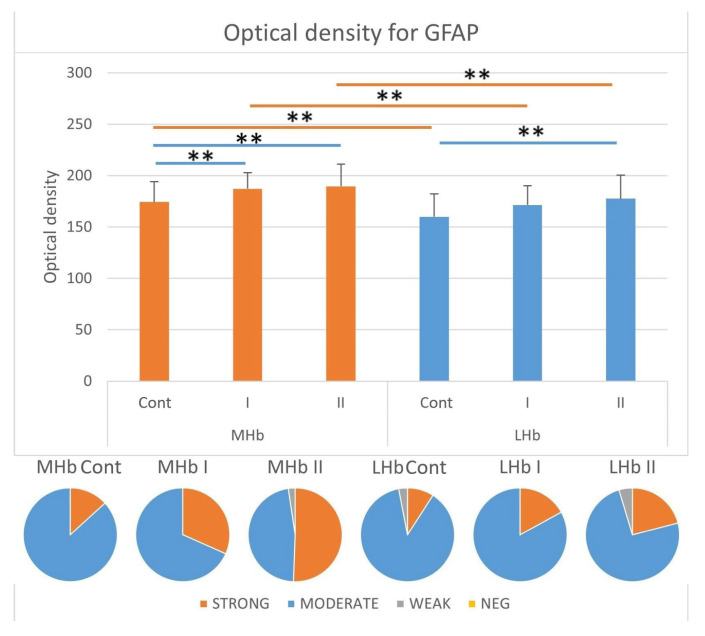
Immunostaining intensity represented as optical density in GFAP-positive cells in the LHb and MHb parts of the habenula. Pie charts demonstrate the ratio of cells recognized as strong, moderate, weak, and negative in all studied groups. **, statistically significant differences between the studied groups and between parts of the Hb within the groups, *p* < 0.05, Kruskal–Wallis.

Analyses of the intensity of immunohistochemical reactions in GFAP-positive astrocytes showed statistically significantly higher optical density (OD) values in the MHb compared to the LHb in individual experimental groups (Figure 6).

In the MHb, the immunostaining intensity of cells increased in individuals receiving MSG (I and II) compared to control animals (Cont). In groups Cont and I, most astrocytes were characterized by a moderate intensity of reaction for GFAP (approx. 87% and approx. 68% of tested cells, respectively). The remaining cells immunopositive for the tested protein showed a strong intensity of immunostaining for GFAP (group Cont, approx. 13%; group I, approx. 32% of the studied cells). In rats receiving 4 g/kg b.w. MSG (II) among GFAP-IR astrocytes, the strong staining was characteristic for approx. 51% of cells, about 47% were the astrocytes with moderate intensity, and the remaining cells were characterized by a weak reaction intensity for the tested protein (approx. 2% of the cells) (Figure 6).

In the LHb, the immunostaining intensity of cells with GFAP immunoexpression increased in individuals receiving MSG at a high dose (II) compared to control animals (Cont). In all individuals, most astrocytes were characterized by a moderate reaction intensity for the studied protein. These cells among GFAP-IR astrocytes in group Cont constituted about 88%; in group I, about 83%; and in group II, about 74%. The least strongly stained cells were present in control animals (approx. 9% of cells). In rats receiving MSG, the percentage of strongly stained cells was about 17% in group I and about 21% in group II. The remaining cells immunopositive for the tested protein in groups Cont and II were characterized by a weak reaction intensity for GFAP (group Cont, approx. 3%; group II, 5%) (Figure 6).

#### 3.3.5. Immunostaining Intensity of the Cytoplasm of S100β-IR Cells

**Figure 7:**
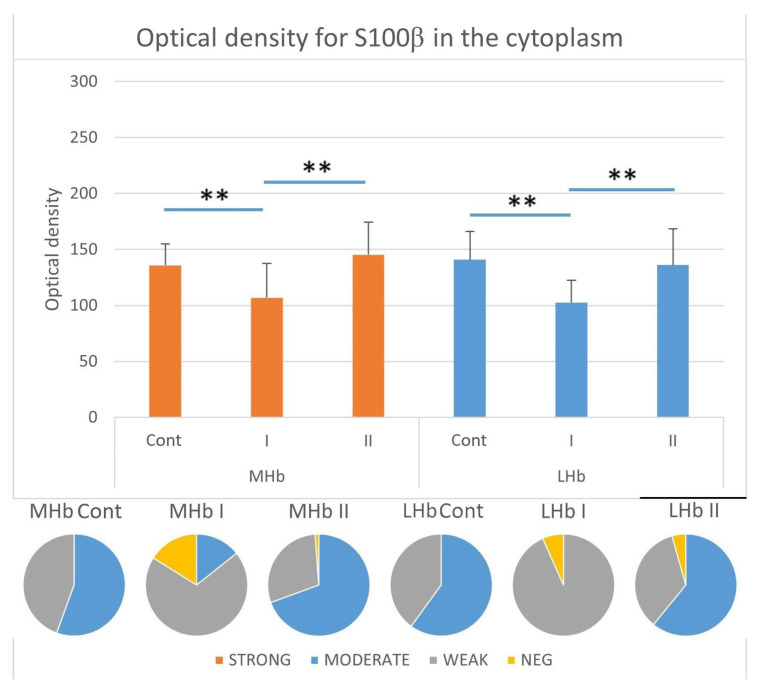
Immunostaining intensity represented as optical density in the cytoplasm of S100β-positive cells in the LHb and MHb parts of the habenula. Pie charts demonstrate the ratio of cells recognized as strong, moderate, weak, and negative in all studied groups. **, statistically significant differences between the studied groups and between parts of the Hb within the groups, *p* < 0.05, Kruskal–Wallis.

Analyses of cytoplasmic reaction intensity in S100β-positive astrocytes did not show statistically significant differences in optical density (OD) values between the studied parts of the Hb in the individual experimental groups (Figure 7).

In the MHb, the cytoplasmic immunostaining intensity for S100β decreased in individuals receiving MSG at a dose of 2 g/kg b.w. (I) compared to control animals (Cont) and animals from group II in which most astrocytes were characterized by moderate cytoplasmic reaction intensity for the tested protein (group Cont, approx. 56%; group II, approx. 69% S100β-IR astrocytes). The percentage of moderately stained cells in rats from group I was lower and amounted to approx. 14%. In this group, the most numerous were astrocytes with weak cytoplasmic immunostaining intensity for S100β (approx. 70%), while in control animals and those receiving MSG at a dose of 4 g/kg b.w. (II) they constituted approx. 44% and approx. 29%, respectively. In addition, in rats from groups I and II, cells recognized as negative for the studied protein were also shown. Their percentage was approx. 16% and approx. 1%, respectively, among all S100β-positive astrocytes (Figure 7).

In the LHb, similarly to the MHb, the cytoplasmic immunostaining intensity for S100β decreased in individuals receiving MSG at a dose of 2 g/kg b.w. (I) compared to control animals (Cont) and animals from group II, in which the majority of astrocytes were characterized by a moderate intensity of reaction (approx. 60% for group Cont and approx. 61% for group II of S100β-IR astrocytes). In animals from group I, the most numerous astrocytes showed weak cytoplasmic immunostaining intensity for the studied protein (approx. 93% of cells). In control animals and those from group II, weakly stained cells constituted approx. 40% and approx. 34%, respectively. Similarly to the MHb, in all rats receiving MSG, cells recognized as negative for S100β were demonstrated. Their percentage among all immunopositive glial cells was approx. 7% in group I and approx. 4% in group II (Figure 7).

#### 3.3.6. Immunostaining Intensity of the Nucleus of S100β-IR Cells

**Figure 8:**
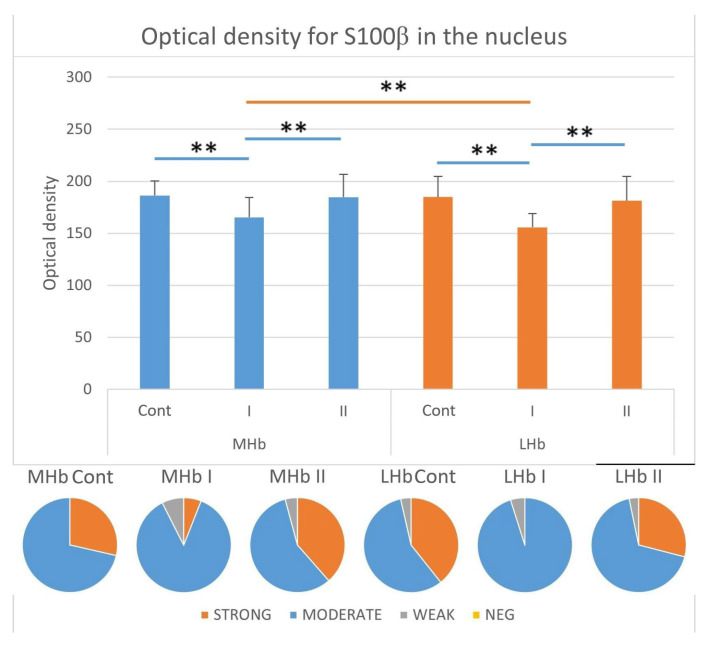
Immunostaining intensity represented as optical density in the nucleus of S100β-positive cells in the LHb and MHb parts of the habenula. Pie charts demonstrate the ratio of cells recognized as strong, moderate, weak, and negative in all studied groups. **, statistically significant differences between the studied groups and between parts of the Hb within the groups, *p* < 0.05, Kruskal–Wallis.

Analyses of the nuclear intensity of the reaction in S100β-positive astrocytes showed differences only in rats from group I between the studied parts of the Hb. Statistically significantly higher optical density (OD) values were observed in the MHb compared to the LHb (Figure 8). In the MHb, the intensity of nuclear immunostaining for S100β in astrocytes decreased in individuals receiving MSG at a dose of 2 g/kg b.w. compared to control animals (Cont) and those in group II. In all studied rats, most astrocytes were characterized by moderate nuclear immunostaining intensity for S100β. The percentage of these cells in group Cont was approx. 71%; in group I, approx. 87%; and in group II, approx. 57% of all S100β-IR cells. In control animals (Cont), the remaining astrocytes (approx. 29%) showed strong immunostaining intensity in the cell nuclei. In individuals from group I, such glia constituted approx. 6%, while in rats receiving MSG at a dose of 4 g/kg b.w. (II) they constituted approx. 38% of all S100β-IR cells. In addition, in rats from groups I and II, there were also astrocytes with weak nuclear immunostaining intensity for the studied protein. The percentage of such glia was approx. 7% in group I and approx. 4% in group II among all S100β-IR cells (Figure 8).

In the LHb, similarly to the MHb, the immunostaining intensity for S100β cell nuclei in astrocytes decreased in individuals receiving MSG at a dose of 2 g/kg b.w. (I) compared to control animals (Cont) and to animals from group II. In all rats, most astrocytes were characterized by moderate nuclear immunostaining intensity for the tested protein. Such glia constituted about 57% in group Cont, about 95% in group I, and about 68% in group II of all S100β-IR astrocytes. Cells with strongly stained nuclei were most numerous in animals from the control group (Cont) where their percentage was about 39% positive glial cells compared to group I (0%) and group II (about 29%). The remaining astrocytes immunostained for S100β were characterized by weak nuclear reaction intensity for the studied protein. These cells constituted about 4% in group Cont, about 5% in group I, and about 3% in group II (Figure 8).

## 4. Discussion

In the presented study, astroglia reactive for GFAP and S100β was demonstrated in the MHb and LHb after subcutaneous administration of MSG to adult male rats. Female rats in which GFAP immunoexpression in astrocytes is influenced by sex steroid hormones were not used in the experiment [54].

In our studies, we showed that in the MHb, the number of GFAP-positive cells was higher in relation to the LHb in all studied groups. On the other hand, the number of S100β-IR cells in both parts of the Hb was comparable. Similar results were obtained by other authors, who showed in mice that the level of GFAP expression in the LHb is significantly lower than in the MHb, and S100β proteins are maintained at a similar level in the entire Hb [7]. Moreover, in both parts of the Hb, in animals from groups I and II, we observed cells immunopositive for the studied proteins with altered morphology, characterized by hypertrophy of cellular bodies and processes. The most intense reaction was shown by GFAP-positive cells located in the MHb of animals from group II. This may indicate a different degree of lesion intensity depending on the part of the Hb and the dose of MSG administered to rats. Cells immunopositive for S100β protein in the Hb of rats from groups I and II also showed signs of immunoreactivity. However, a slight hypertrophy of their bodies was observed in comparison to GFAP-IR astrocytes. Similar reactive behavior of astroglia was demonstrated in other brain areas of rats receiving MSG [44,45,46].

In the MHb, changes in the morphology of the studied glia were accompanied by an increase in the number of astrocytes with GFAP immunoexpression independent of the dose of MSG administered to rats in comparison to control animals. Moreover, in rats from group I, a higher number of S100β-positive cells was observed compared to individuals from groups Cont and II. At the same time, among GFAP-IR glia, an increasing percentage of cells with strong cytoplasmic immunostaining for the studied protein was observed along with the increasing dose of MSG compared to the control group (Cont). The above-described astroglial reactivity most likely occurs secondary to the increase in extracellular Glu concentration. Increased GFAP immunoexpression in astroglia may be related to the need to enhance the uptake of this neurotransmitter from the perineuronal space. This protein plays a key role in modulating the transport and function of glutamate-transporting proteins [58]. Specific Na+-dependent membrane transporters, i.e., glutamate–aspartate transporter (GLAST) and glutamate transporter-1 (GLT-1), are located in perisynaptic astrocytic processes. These cells uptake about 90% of Glu from the extracellular space. This process is mainly mediated by GLT-1 transporters [6,34,59]. In the states of hyperexcitation of neurons, the activity of the transporters is enhanced and there is increased GLT-1 expression, which immediately affects the reduction in extracellular Glu levels in order to protect neurons from damage [34]. It has been shown in an adult mouse model of epilepsy that GLT-1 and GLAST immunoreactivity significantly increases in the first 3 days after intrahippocampal injection of kainate acid (NMDA-R analog). However, after 7 days, a significant decrease was observed, which correlated with the occurrence of epileptic seizures in animals [60]. GLT-1 and GLAST play an important role in the regulation of neuronal excitation in the Hb [6]. Glutamatergic neurons constitute the main cell population in both the MHb and LHb. In addition, the presence of inhibitory neurons for which Glu is a direct substrate for the production of ℽ-ammobutyric acid (GABA) was found in the LHb [1,2,61]. In this part, a significantly greater number of transporters for Glu was also demonstrated in comparison to the MHb. The dominant one was GLT-1, which was confirmed by examining the expression of GLT-1 and GLAST mRNA [6,7]. In addition, other authors demonstrated the colocalization of GLT-1 with astroglial intermediate filaments, as well as the participation of the cytoskeleton in the movement of this transporter along astrocytic processes [62]. GFAP also participates in the anchoring of GLAST in the cellular membrane [63]. Its increased expression was demonstrated in cultured astrocytes maintained in conditions of increased Glu concentration [64,65].

The increase in the number of GFAP-immunopositive cells that was revealed in our studies may result from several processes. Firstly, there may be an increased production of the tested protein in astrocytes that were previously immunonegative. GFAP is a marker for almost all reactive but not all non-reactive astrocytes. They have GFAP at a level that is undetectable by immunohistochemical methods [66]. Such behavior of astroglia is observed in the course of mild or moderate astrogliosis. Then, there is a clearly increased immunoexpression of GFAP, as well as hypertrophy of almost all astrocytes. The increase in the number of cells immunopositive for the tested protein may result from the proliferation of the tested glia. In rats, intrastriatal and intrahypothalamic injections of quinolinic acid, an endogenous excitotoxin that acts as a glutamate agonist at NMDA receptors, led to the appearance of reactive and proliferating astroglia. Astrocyte reactivity typically develops several days after exposure to the damaging factor. Initially, there is an activation and proliferation of microglia, which react even to the smallest pathological changes occurring in the environment [67,68].

Microglia, which can be activated by a high level of glutamate, may be the source of mitogenic and morphogenic substances acting on astrocytes [15,20]. The reactive behavior of astroglia is induced by numerous proinflammatory cytokines and chemokines. Astrocytes express receptors, among others, for interleukin 1β (IL-1β) and tumor necrosis factor (TNFα) [20,34]. Activation of astrocytic receptors with a recombinant form of IL-1β injected into the cerebral cortex of adult rats resulted in astrocyte stimulation and an increase in the number of GFAP-positive cells. Moreover, an increase in the activity of glutamine synthetase in astroglia, one of the key enzymes involved in intracellular glutamate metabolism, was demonstrated [69]. Other authors, however, observed a positive correlation between the presence of TNFα and the increase in GFAP expression in astrocytes in the brain [70]. IL-1β and TNFα induce a proinflammatory response in astrocytes. In addition, primary cultured astrocytes in the presence of IL-1β produce TNFα themselves. This compound activates a signaling pathway dependent on the nuclear transcription factor kappa B (NF-κB), which in turn leads to the inhibition of Glu uptake by astrocytes and contributes to an increase in its concentration in the extracellular space [10,15,71].

The cells with immunoexpression of S100β observed in the MHb and LHb of animals receiving MSG had more numerous and longer processes with a brown reaction product. This fact may be related to excessive stimulation of membrane glutamatergic NMDA receptors located in astrocytic processes and the bodies of the cells. As a result of their activation, there is an increased intracellular influx of Ca^2+^ ions, which triggers mechanisms in astrocytes that aim to reduce their intracellular level. S100β fulfils such a function as a buffering protein. In addition, S100β is involved in calcium-dependent intracellular processes, including an influence on the activity and metabolism of many enzymes, phosphorylation of various proteins, and gene transcription, and it participates in the processes of cell proliferation and differentiation. S100β also increases the frequency of mitotic divisions by remodeling the cytoskeleton of cells. Interactions of this protein with GFAP affect the cell cycle, growth, and differentiation of astrocytes [72,73,74].

In the MHb in rats from group I, the changes in the morphology of the studied glia were accompanied by an increase in the number of glial cells with S100β immunoexpression and a simultaneous decrease in the cytoplasmic-nuclear immunostaining intensity. This phenomenon may result from the active secretion of S100β into the extracellular space, where it acts in an autocrine and paracrine manner on neurons and other glial cells [72,73,74]. Astrocytes release this protein under the influence of various factors, e.g., those produced by damaged neurons but also by stimulation of group II metabotropic glutamate receptors (mGluR3), which has been demonstrated in the mouse hippocampus in a model of epilepsy [75,76]. S100β present in the extracellular space modulates glutamate uptake by astrocytes and also stimulates glia to divide, protecting nerve cells from excitotoxic damage. Moreover, extracellularly located S-100β also affects neurons, which has been shown in numerous in vitro studies. At low concentrations, it protects rat hippocampal nerve cells from excitotoxicity induced by activation of NMDA receptors [72,73]. However, at higher concentrations (micromolar), S100β is toxic and can lead to apoptosis of nerve cells, as well as to the activation of microglia. This protein increases the synthesis of IL-1β and TNFα in microglial cells showing synergistic effects together with them [72,73,77]. Based on this literature data, increased secretion of S100β may be the reason for the comparable number of cells immunopositive for the tested protein in the MHb of animals receiving MSG at a high dose (group II) compared to the control individuals. However, in the LHb of rats from groups I and II, the lack of an increase in the number of S100β-positive astrocytes, along with an unchanged number of GFAP-positive cells, may be explained by cytotoxic damage to these cells caused by MSG administration. Studies conducted on mice and rats have shown that MSG, through the excessive activation of specific receptors, may lead to increased production of reactive oxygen species, activation of many enzymes, and apoptotic cell death [78,79]. This fact could be confirmed by the results of microscopic and morphometric analyses of the cerebellum in rats after intragastric administration of MSG for 14 days at a dose of 3 g/kg b.w. and in combination with ascorbic acid (vitamin C) with antioxidant properties. In animals receiving only MSG, no increase in the number of GFAP-positive cells was observed, which was explained by their damage. However, in rats receiving MSG in combination with vitamin C, a significant increase in GFAP immunoreactivity in astrocytes was observed [46].

One of the limitations of this study is the short, three-day observation period following the administration of monosodium glutamate (MSG), which was determined based on data from the literature [42]. The short-time duration of the experiment allows for the detection of only the early glial response and does not provide a basis for drawing conclusions about long-term consequences, such as chronic inflammatory processes or neuro-degeneration. However, we believe that our results are still meaningful, as early glial alterations, such as changes in astrocyte reactivity and distribution, can serve as initial indicators of subsequent pathological processes. Another limitation of the present study is the lack of direct measurements of glutamate levels in the Hb. Although the observed morphological changes and glial activation may suggest the involvement of excitotoxic mechanisms, glutamate concentrations were not assessed, which would be necessary to confirm a direct relationship between MSG administration and excitotoxicity in this brain region. In future studies, we plan to extend the observation period as well as expand our analyses to include the evaluation of glutamatergic neurotransmission, which will allow for a more precise understanding of the mechanisms underlying the observed effects, in order to fully assess the impact of monosodium glutamate on the central nervous system.

## 5. Conclusions

Our results revealed that MSG leads to astroglia reactivity in the Hb. In the MHb of rats receiving this compound, especially at a high dose, hypertrophy of GFAP-IR and S100β-IR cells was demonstrated. These changes were accompanied by an increase in the number of astrocytes with immunoexpression of the studied proteins, which in the case of S100β-positive cells was found only in rats from group I. In the LHb, only hypertrophy of processes in S100β-IR glia was observed. Moreover, in both parts of the Hb, GFAP-IR astrocytes were characterized, mainly in group II, by an increase in the cytoplasmic immunostaining intensity. In animals from group I, S100β-IR cells showed a reduced nuclear–cytoplasmic immunostaining intensity. We suggest that the observed changes in the MHb may reflect a phenotypic transformation of previously non-reactive astrocytes, accompanied by increased immunoexpression of the studied proteins. However, the possibility of astroglial proliferation cannot be excluded. In the LHb, increased secretion of S100β into the extracellular space from existing astrocytes may occur, as well as excitotoxic glial damage, particularly in rats receiving MSG at a dose of 4 g/kg body weight.

The simultaneous occurrence of astrocyte proliferation in the MHb and excitotoxic damage in the LHb may be explained by regional differences in astrocyte properties. Astrocytes in the MHb may have greater resistance to glutamate-induced injury and instead initiate reactive and proliferative responses. In contrast, LHb astrocytes may be more vulnerable to excitotoxicity, resulting in structural damage rather than proliferation. This divergence highlights the functional and molecular heterogeneity of astroglia within the brain. Understanding the reaction of astroglia in conditions of increased Glu concentration may in the future be the basis for the development of new therapies in the treatment of neurological diseases that develop as a result of improper functioning of the Hb. For this purpose, it seems crucial to undertake further research aimed first at identifying the subtype of reactive astrocytes and their interactions with surrounding structures, and subsequently at elucidating the mechanisms that inhibit their negative activity or enhance their protective functions, which in later stages of research may contribute to increased neuronal survival.

## Figures and Tables

**Table 1:** Morphometric parameters of GFAP-IR astrocytes in the LHb and MHb parts of habenula.

GFAP-IR Cells						
	MHb			LHb		
	Cont	I	II	Cont	I	II
Processes number	2.24 ± 0.98 ^a^	3.15 ± 1.09 ^b^	4.25 ± 1.3 ^c^	2.08 ± 1.04	2.41 ± 1.1 *	2.07 ± 0.98 *
Processes length	5.66 ± 2.09 ^d^	8.48 ± 3.44 ^e^	11.68 ± 4.47 ^f^	5.82 ± 2.36	7.27 ± 3.53 ^(^**^)^	6.99 ± 3.02 **
Area	25.16 ± 5.53 ^d^	37.63 ± 8.37 ^e^	44.66± 11.86 ^f^	26.32 ± 6.9	27.96 ± 8.14 **	28.64 ± 7.4 **
Perimeter	19.48 ± 2.63 ^d^	23.83 ± 2.89 ^e^	25.95 ± 3.48 ^f^	20.14 ± 2.58	21.41 ± 3.44 **	21.09 ± 2.5 **
Shape index	0.83 ± 0.1	0.83 ± 0.09	0.82 ± 0.09	0.8 ± 0.06 ^(ab)^	0.76 ± 0.08 ^(a)^*	0.8 ± 0.09 ^(b)^

Different letters represent statistical differences between groups (a, b, c—ANOVA, *p* < 0.01; (a, b, c)—ANOVA, *p* < 0.05; d, e, f—Kruskal–Wallis, *p* < 0.01; (d, e, f)—Kruskal–Wallis, *p* < 0.05). *, statistical differences between parts of the habenula (LHb vs. MHb) within the groups, ANOVA *p* < 0.01; **, statistical differences between parts of the habenula (LHb vs. MHb) within the groups, Kruskal–Wallis *p* < 0.01; (**), statistical differences between parts of the habenula (LHb vs. MHb) within the groups, Kruskal–Wallis *p* < 0.05.

**Table 2:** Morphometric parameters of S100β-IR astrocytes in the LHb and MHb parts of the habenula.

S100β-IR Cells						
	MHb			LHb		
	Cont	I	II	Cont	I	II
Processes number	0.8 ± 1.02 ^a^	1.42 ± 1.15 ^b^	2.32 ± 1.56 ^c^	0.33 ± 0.56 ^a^*	0.69 ± 0.66 ^b^*	0.81 ± 0.68 ^b^*
Processes length	1.01 ± 1.42 ^d^	5.13 ± 2.6 ^e^	5.19 ± 3.32 ^e^	0.64 ± 1.43 ^d^	1.3 ± 1.43 ^(e)^**	1.97 ± 2.03 ^e^**
Area	19.21 ± 4.21 ^d^	27.01 ± 6.9 ^e^	27.57 ± 7.87 ^e^	21.46± 4.97 ^(^**^)^	21.1 ± 4.49 **	21.84 ± 5.93 **
Perimeter	17.21 ± 1.89 ^d^	20.64 ± 2.72 ^e^	20.78 ± 2.98 ^e^	18.46± 2.36 ^(^**^)^	18.21 ± 2.28 **	18.86 ± 2.76 **
Shape index	0.81 ± 0.08	0.79 ± 0.08	0.79 ± 0.09	0.79 ± 0.08	0.8 ± 0.09	0.76 ± 0.09

Different letters represent statistical differences between groups (a, b, c—ANOVA, *p* < 0.01; (a, b, c)—ANOVA, *p* < 0.05; d, e, f—Kruskal–Wallis, *p* < 0.01; (d, e, f)—Kruskal–Wallis, *p* < 0.05). *, statistical differences between parts of the habenula (LHb vs. MHb) within the groups, ANOVA *p* < 0.01; **, statistical differences between parts of the habenula (LHb vs. MHb) within the groups, Kruskal–Wallis *p* < 0.01; (**), statistical differences between parts of the habenula (LHb vs. MHb) within the groups, Krus-kal-Wallis *p* < 0.05.

## Data Availability

Data are contained within the article.

## Abbreviations

Hb	Habenula
MHb	Medial part of habenula
LHb	Lateral part of habenula
CNS	Central nervous system
Glu	Glutamate
MSG	Monosodium glutamate
GFAP	Glial fibrillary acidic protein
GFAP-IR	Immunoreactive for glial fibrillary acidic protein
S100β-IR	Immunoreactive for S100β protein
NMDA-R	N-methyl-D-aspartate receptor
RT	Room temperature
DAB	3,3′-diaminobenzidine tetrachloride
Si	Shape index
OD	Optical density
ANOVA	One-way analysis of variance
GLAST	Glutamate-aspartate transporter
GLT-1	Glutamate transporter-1
GABA	ℽ-ammobutyric acid
TNFα	Tumor necrosis factor
IL-1β	Interleukin 1β
NF-κB	Nuclear transcription factor kappa B
